# Use of Chinese medicine by cancer patients: a review of surveys

**DOI:** 10.1186/1749-8546-6-22

**Published:** 2011-06-09

**Authors:** Bridget Carmady, Caroline A Smith

**Affiliations:** 1 Centre for Complementary Medicine Research, University of Western Sydney, Locked Bag 1797, Penrith South DC 2751, New South Wales, Australia

## Abstract

Chinese medicine has been used to treat a variety of cancer-related conditions. This study aims to examine the prevalence and patterns of Chinese medicine usage by cancer patients. We reviewed articles written in English and found only the Chinese medicine usage from the studies on complementary and alternative medicine (CAM). Seventy four (74) out of 81 articles reported rates of CAM usage ranging from 2.6 to 100%. Acupuncture was reported in 71 out of 81 studies. Other less commonly reported modalities included *Qigong *(*n *= 17), Chinese herbal medicine (*n *= 11), Taichi (*n *= 10), acupressure (*n *= 6), moxibustion (*n *= 2), Chinese dietary therapy (*n *= 1), Chinese massage (*n *= 1), cupping (*n *= 1) and other Chinese medicine modalities (*n *= 19). This review also found important limitations of the English language articles on CAM usage in cancer patients. Our results show that Chinese medicine, in particular Chinese herbal medicine, is commonly used by cancer patients. Further research is warranted to include studies not written in English.

## Background

Conventional cancer treatments such as chemotherapy and radiation therapy have shown some effectiveness for reducing or eradicating cancers; however, they can produce unpleasant side effects, e.g. nausea, vomiting, changes in bowel habits, fatigue and hair loss. Chinese medicine is increasingly used as an adjunctive treatment option for cancer patients and a way of reducing or managing side effects of conventional cancer treatment.

Chinese medicinal herbs such as *Ginkgo biloba *has been reported to have chemo-preventive activities for treating certain cancers such as ovarian, breast and brain [1]. Acupuncture is being used to relieve side effects of conventional cancer treatment. While some laboratory and clinical research found some immune boosting capabilities of acupuncture in cancer patients [2,3], most clinical research has focused on symptom management, in particular, the management of chemotherapy induced nausea and vomiting [4-6].

This study reviews the articles published in English language complementary and alternative medicine (CAM) literature on the prevalence and patterns of Chinese medicine usage by cancer patients and informs patients, researchers, health care providers and policy makers of the current use of Chinese medicine in the CAM context.

## Methods

### Literature search

Our working definition of CAM was an inclusive term incorporating both complementary medicine and therapies (modalities and/or systems), namely the concepts of health and medical systems, practices and products not currently recognised as part of conventional medicine, alternative medicine, traditional medicine (indigenous medicine and practices), and integrative medicine (CAM used alongside with the mainstream medicine) [7]. For the purposes of this review Chinese medicine includes acupuncture, Chinese herbal medicine, remedial massage, exercise and breathing therapy (e.g. *Qigong*) as well as diet and lifestyle advice in primary health care [8].

We searched major databases, namely AMED, CINAHL, PubMED, Science Direct and Cochrane Library, using specific terms to retrieve surveys published in English. One author (BC) screened all the titles and abstracts to identify relevant studies. Survey studies containing prevalence rates for at least one Chinese medicine modality for treating cancer patients were included. Studies on children were not excluded.

### Data extraction

The following data was extracted: country of study, number of study participants, type of study (quantitative, qualitative, mixed), group setting (e.g. hospital, cancer registry), type of cancer, age, gender, ethnicity, marital status, education, prevalence of individual Chinese medicine modality, prevalence, sources of CAM information and reasons for CAM usage.

### Quality-assessment

The quality of the CAM surveys were assessed according to Bishop *et al. *[9], based on the Strengthening the Reporting of Observational Studies in Epidemiology (STROBE) statement [10]. Reported information was assessed with scores which were weighted for importance. Both authors (BC and CAS) scored the included articles. Final scores were consensus of both authors. Four articles [11-14] were primarily qualitative and therefore not assessed. Three items were scored a maximum of two points, eight items one point and six items 0.5 points. The maximum total score was 17.

### Data analysis

We described the general characteristics of users of Chinese medicine including both Chinese medicine specific studies and Chinese medicine embedded within CAM studies. Data was analysed with SPSS Statistics 17.0 (IBM, USA). Descriptive statistics, means, medians, ranges, frequencies and percentages characterised the studies.

## Results

**Figure 1:**
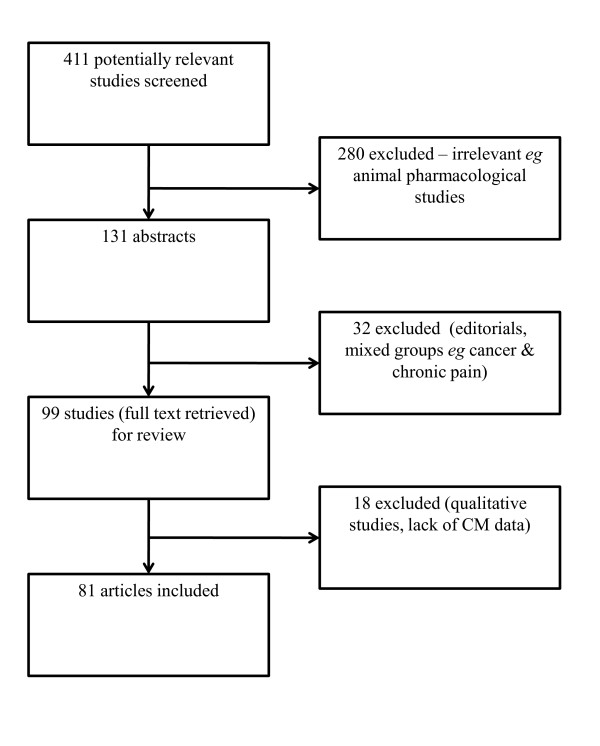
**Process of study identification and selection**.

The search identified a total of 411 studies for screening. Ninety nine screened articles were retrieved for further evaluation. Eighty one studies met the inclusion criteria and were included in this review (Figure 1).

### Characteristics of the studies

The included 81 studies spanned a period of 15 years (Table 1), with the majority published in the last five years (60.5%). While the surveys were carried out around the world, a large number of surveys were conducted in North America, the United States (US) in particular (33.3%). Sample sizes of the studies ranged from 16 to 22,352 with a median of 189 participants. Two thirds of the participants were female (66.7%). Participants had a mean age of 56.0 ± 11.9 years (mean ± standard deviation, SD) (ranging from 3 to 71 years), were married or in a *de facto *marriage (70.6%) and had completed high school education (35.8%). The majority (84.5%) were of Caucasian ethnicity. Survey participants were recruited from hospital settings including outpatient clinics, cancer institutes and palliative care (70.4%), with convenience sampling (61%). Participants had a range of cancers (49.4%); however, a significant focus was on women with breast cancer (25.9%). Most studies used a self-administered questionnaire (52%).

**Table 1:** Characteristics of included studies (*n *= 81).

	Number of studies	%
Year (in 5-year blocks)		
2010	7	8.6
2009-2005	49	60.5
2004-2000	22	27.2
1999-1995	3	3.7
Country (by region)		
North America	35	43.2
UK & Europe	20	24.7
Asia	19	23.5
Middle East	3	3.7
Australia and New Zealand	3	3.7
Multiple	1	1.2
Country or region (by individual countries or regions)		
US	27	33.3
Canada	8	9.9
UK	7	8.6
Europe	6	7.4
Taiwan	4	4.9
Other individual countries	29	35.9
Group setting		
Hospital including outpatient clinics, cancer institutes, palliative care	57	70.4
Cancer/tumour registry	12	14.8
General Population	6	7.4
Other	6	7.4
Data Collection Methods		
Self-report questionnaire	42	51.9
Interviewer led (in person or telephone)	37	45.7
Database e.g. insurance claims	2	2.5
Sampling method		
Random	18	22.2
Systematic	6	7.4
Stratified	7	8.6
Convenience	50	61.7
Cancer type		
Various (mixed cancers within groups)	40	49.4
Breast	21	25.9
Prostate	5	6.2
Other	15	18.5
Gender, mean %		
Female		66.7
Male		33.2
Age, mean (Missing data = 31)	56.0	
Marital status, mean % (Missing data = 34)		
Married or de-facto		70.6
Other		29.4
Education, mean % (Missing data = 27)		
Primary School		12.3
High School		35.8
University		18.5

### Prevalence of Chinese medicine use

Seventy four studies reported the rates of CAM usage which ranged from 2.6 to 100%. Acupuncture was the most frequently reported Chinese medicine modality included within CAM. A total of 71 studies reported data on acupuncture. Other less commonly reported modalities included *Qigong*, Chinese herbal medicine, Taichi, acupressure, moxibustion, Chinese dietary therapy, Chinese massage and cupping.

We examined the prevalence of Chinese medicine usage and reported the range and a mean prevalence (Table 2). Chinese herbal medicine was the most frequently used modality within Chinese medicine; however data were only available from 11 of the 81 studies. Usage ranged from a low prevalence of 0.7% to a high prevalence of 94.4%, with an average use rate of 35.6%. Acupuncture prevalence ranged from 0.2 to 17.1% with a mean of 4.5% extracted from 71 studies. Usage of *Qigong *by cancer patients was reported in 17 studies with a mean prevalence rate of 12.7%. Usage reported in these studies ranged from 0.4 to 100%. Taichi prevalence ranged from 1.7 to 40.6% reported in ten studies with a mean of 9.0%. Other Chinese medicine modalities (acupressure, Chinese dietary therapies, Chinese massage, moxibustion and cupping) were reported with few data in the 11 studies covering these modalities (Table 2). Mixed Chinese medicine prevalence rates (where cancer patients reported using several Chinese modalities concurrently) were also reported. Nineteen of the studies reported such data with a mean prevalence of 17.8% (ranging from 0.3 to 100%).

**Table 2:** Prevalence of CAM and Chinese Medicine usage

Modality (No. of studies)	Prevalence Mean %	SD	Prevalence Min %	Prevalence Max %
CAM (74)	49.3	24.5	2.6	100
Chinese herbal medicine (11)	35.6	42.1	0.7	94.4
Mixed Chinese medicine (19)	17.8	28.6	0.3	100
Chinese dietary therapy (1)	16.7	-	16.7	16.7
*Qigong *(17)	12.7	25.2	0.4	100
Taichi (10)	9.0	11.5	1.7	40.6
Acupressure (6)	6.3	6.4	1.1	18.8
Acupuncture (71)	4.5	3.8	0.2	17.1
Moxibustion (2)	2.4	1.1	1.7	3.2
Chinese massage (1)	2.4	-	2.4	2.4
Cupping (1)	1.3	-	1.3	1.3

### Use patterns of Chinese medicine modalities

Our search identified nine studies that provided detailed data on the usage patterns of Chinese medicine [11,15-22]. The aims of these studies were quite diverse. We were not able to provide a systematic summary of these data but a narrative summary.

Studies examining patterns of Chinese medicine usage varied in study design. One study used qualitative methods [11]; another study used a retrospective analysis of insurance registration and claim datasets [20], and seven studies were questionnaire-based surveys [15-19,21,22]. All seven surveys included Chinese or other Asian populations (Mainland China, Taiwan, Hong Kong, Singapore), or Chinese immigrants in Canada. Seven studies reported an overall Chinese medicine usage rate attributed to Chinese medicinal herbs, *Qigong*, acupuncture and moxibustion.

Within the nine studies, usage of Chinese medicinal herbs varied widely; however the majority reported high usage of 94.4% [19], 93.75% [11], 86.4% [17], 76.75% [15] and one low rate of 2.48% [20]. Examples are presented in the following studies. Shih *et al. *[22] reported additional details on the types of Chinese medicinal herbs and related modalities in particular food supplements. Forty five percent of participants used bird (swallow) nests and 28.6% chicken essence; 53% used prescribed herbs, of which 15.4% used Lingzhi, and 8% used Chinese herbal formulae. In the study by Xu *et al. *[11], 50% of participants used individually tailored herbs, 6% standard herbal formulae and 38% both types. Xu *et al*. reported that all participants (*n *= 16) practiced *Qigong*.

### Characteristics of Chinese medicine users

Three [17,18,21] of the nine studies reported the characteristics of Chinese medicine users. Pu *et al. *[21] surveyed 2034 patients with cervical, breast, lung, liver and colorectal cancers and highlighted patients' usage of Chinese medicine modalities according to cancer types. Chinese medicine as a broad modality was more likely to be used by patients with breast, lung, liver and colorectal cancers whereas acupuncture was more likely to be used by liver and colorectal patients. Pu *et al*. examined the correlation of socio-economic factors (e.g. religion, education and income) with Chinese medicine usage. While more Buddhists used Chinese medicine, acupuncture usage was not distinctive in patients with any religion. Acupuncture users were mostly female cancer patients with higher education. According to the study by Pu *et al*., participants earning a higher income were about 52% more likely than lower income groups to use Chinese medicine. Similarly, Cui *et al. *[17] found that more participants with a higher education and higher income used Chinese herbal medicine. Ferro *et al. *[18] found that Chinese medicine was used by less acculturated patients twice as much as acculturated patients.

### Motivation to use and the perceived effectiveness of Chinese medicine

Motivation to use and the perceived effectiveness of Chinese medicine modalities were reported in three studies [11,15,17]. Xu *et al. *[11] highlighted four important reasons for Chinese medicine usage among 28 Chinese cancer patients: (1) Chinese medicine as a popular and culturally acceptable process of self-help, (2) fear of chemotherapy damaging the vital essence, (3) importance of individualised prescriptions and (4) empowerment with self-help. Almost all participants used Chinese medicine to avoid or reduce adverse effects from cancer treatment. Overall, health benefits, quality of life and ability to function were significantly improved with Chinese medicine. Benefits attributed to Chinese medicine included reduced fatigue, nausea and vomiting, constipation, stress, weakness and weight gain.

Cui *et al. *[17] found that the most common reason for using Chinese herbal medicine among breast cancer patients was cancer treatment (81.5%), followed by immune system enhancement (12%), metastasis prevention or side effect management (7.9%), and the reduction of menopausal symptoms (4.7%). Chinese herbal medicine was perceived to be effective or very effective for cancer treatment (78.7%), and 77% of female patients perceived Chinese medicine to be very effective or effective for immune system enhancement. Similar levels of effectiveness were reported for metastasis management and the reduction of menopausal symptoms. Acupuncture, on the other hand, was reported to be less effective with only 48.1% of users considering it to be effective. Chen *et al. *[15] found far more sceptical views among breast cancer patients with only 52% of patients perceiving Chinese herbal medicine as effective and 4% as very effective in assisting cancer treatment.

### Study quality

Overal study quality (Additional file 1) was scored between 32 and 94%, with 95% of studies scoring above a 50% threshold for the 77 quantitiave studies [15-91]. Fourty four studies omitted piloting of instruments. Fourty seven studies used convenience sampling. Only eight studies reported non-response bias. Overall measures of socio-economic status were included and reported. All studies reported prevalence but many failed to examine the reasons for usage. Many cancer studies (*n *= 11) reported the usage starting from the time of diagnosis, thereby omitting patterns of usage prior to diagnosis.

## Discussion

Acupuncture was the most frequently reported Chinese medicine modality with nearly 90% of the studies containing prevalence data. However, among more comprehensive studies of Chinese medicine modalities, Chinese herbal medicine was the most commonly used form of Chinese medicine.

Increasing prevalence of CAM usage by cancer patients reflects the growing use of CAM over time [92]. Our review suggests a higher CAM prevalence compared with a prevalence of 31.4%, and a range of 7-64%, reported by Ernst [93]. However, unlike Ernst, we were unable to access non-English language publications.

Major limitations of the studies on the use of Chinese medicine in relation to cancer are as follows. Firstly, non-English language studies, in particular those written in Chinese, were not reviewed and should be included in future studies. Moreover, the inability to access the EMBASE database might have excluded some English language reports. Secondly, the variation in the wide range of CAM use is likely explainable by different cultural contexts, understandings and definitions of what constitutes CAM. Thirdly, incomplete reporting of the definition of CAM adopted by many studies, and the lack of rationale for selecting Chinese medicine modalities were not uncommon. Furthermore, extensive demographic characteristics and related details were not reported. Sampling of the participant population and the generalisability of the findings was not justified. Fourthly, qualitative research accompanied by cross sectional and longitudinal surveys and additional information about cultural and ethnic populations was insufficient for cross cultural comparisons. Further studies should address these limitations.

## Conclusion

Our results show that Chinese medicine, in particular Chinese herbal medicine, is commonly used by cancer patients. Further research is warranted to include studies not written in English.

## Abbreviations

CAM: complementary and alternative medicine; STROBE: Strengthening the Reporting of Observational Studies in Epidemiology; US: United States; SD: standard deviation

## Competing interests

The authors declare that they have no competing interests.

## Authors' contributions

BC searched the databases, performed statistical analysis and drafted the manuscript. CAS conceived the study and drafted the manuscript. Both authors read and approved the final version of the manuscript.

## Supplementary Material

Additional file 1 **Summary of QAT Scores (*n *= 77)**. Summary of QAT Scores Click here for file
