# Supplementary material for: Use of Chinese medicine by cancer patients: a review of surveys
Source: Chin Med. 2011 Jun 9;6:22. doi: 10.1186/1749-8546-6-22 (PMC3148205; doi:10.1186/1749-8546-6-22)
Supplement: Additional file 1 — Summary of QAT Scores (n = 77). Summary of QAT Scores [file 1749-8546-6-22-S1.DOC]

**Additional file 1- Summary of QAT Scores (*n*=77)**

| **QAT item** | **Brief definition** | **Points awarded** | **Frequency of studies, *n* (%)** | **Reference** |
| --- | --- | --- | --- | --- |
| Study Methods | | | | |
| Recall Bias | | | | |
| Low risk | Prospective/current data collection | 2 | 74 (96) |  |
| Some risk | Retrospective data collection within the previous 12 months | 1 | 3 (4) |  |
| Piloted questionnaire (or interview schedule) | Any pilot, feasibility, pretest, or previous use of study material | 1 | 33 (43) |  |
| Address potential sources of bias | Report efforts to address nonresponsive bias (through comparing responders to non-responders) or information bias (through assessing inter-rater reliability) | 1 | 8 (10) |  |
| Adjust for potential confounders | Any adjustment of confounders in analyses of variables associated with CAM use e.g. using ANCOVA, multiple regression | 1 | 45 (58) |  |
| Sampling | | | | |
| Response rate | Where response rate = (no. of participants in the study / No of people invited to take part) x 100 | 1 | 66 (86) |  |
| Representative sampling strategy | Any attempt to achieve a sample of participants that represents the larger population | 1 | 30 (39) |  |
| Participants’ Characteristics | | | | |
| Specific diagnosis | Report participants’ diagnoses | 1 | 77 (100) |  |
| Indicator of socioeconomic status | Report participants’ socioeconomic status (e.g., income, education, employment etc) | 0.5 | 71 (92) |  |
| Age | Report participants’ ages | 0.5 | 75 (97) | [15-50, 52-83,85-91] |
| Ethnicity | Report participants’ ethnicity | 0.5 | 34 (44) |  |
| Gender | Report participants’ gender | 0.5 | 77 (100) | [15-91] |
| CAM Usage | | | | |
| CAM definition | Information about the definition of CAM/a list of CAM modalities to be surveyed is provided to participants | 2 | 71 (92) |  |
| Use of CAM modalities assessed | Report the prevalence of use of specific CAM modalities | 1 | 77 (100) | [15-91] |
| Frequency/duration of CAM uses | Report how often or for what duration the CAM were/are used by study participants | 1 | 11 (14) |  |
| Reasons for CAM use | Report the reasons for the use of CAM by study participants | 2 | 45 (58) |  |
